# The Application of Use Case Modeling in Designing Medical Imaging Information Systems

**DOI:** 10.5402/2013/530729

**Published:** 2013-11-27

**Authors:** Reza Safdari, Jebraeil Farzi, Marjan Ghazisaeidi, Mahboobeh Mirzaee, Azadeh Goodini

**Affiliations:** Department of Health Information Management, School of Allied Medical Sciences, Tehran University of Medical Sciences, Tehran 09821-141556183, Iran

## Abstract

*Introduction*. The essay at hand is aimed at examining the application of use case modeling in analyzing and designing information systems to support Medical Imaging services. *Methods*. The application of use case modeling in analyzing and designing health information systems was examined using electronic databases (Pubmed, Google scholar) resources and the characteristics of the modeling system and its effect on the development and design of the health information systems were analyzed. *Results*. Analyzing the subject indicated that Provident modeling of health information systems should provide for quick access to many health data resources in a way that patients' data can be used in order to expand distant services and comprehensive Medical Imaging advices. Also these experiences show that progress in the infrastructure development stages through gradual and repeated evolution process of user requirements is stronger and this can lead to a decline in the cycle of requirements engineering process in the design of Medical Imaging information systems. *Conclusion*. Use case modeling approach can be effective in directing the problems of health and Medical Imaging information systems towards understanding, focusing on the start and analysis, better planning, repetition, and control.

## 1. Introduction

### 1.1. Background and Objective

“Use case modeling” is in fact a conceptual model and explains the required behaviors in the framework of related nontechnical and practical terms. For many organizations this model is a real model to show the performance requirements and visible outward behaviors of an information system [1]. Use case modeling simply shows in an information system which does what job and with what aim [2]. This technique has been developed as a part of UML in the form of a standard objective-oriented modeling language [3] which is based on the system's simple structure and direct understanding, usually presenting considerable details about the interaction between a system and its users in a structured story-like manner [4].

Use case modeling can be done during software development so that the practical behavior of large systems in their interaction with several users having different requirements is shown [5]. Usually one environment is used in relation to modeling a process [6]. Use case modeling can divide every collection into two particular groups: the system and the factors interacting with this system [7, 8]. These factors are called actors. Actors are not limited to the real people. They can be other systems, equipment, or any other thing which can interact or perform a function in the system [9]. For example, the actors of a radiology use case model can be the radiologists, doctors, patients, technicians, students, researchers, or even Picture Archiving and Communication System (PACS) or Health Information System (HIS) [10]. Considering all the actors interacting with a system and through user cases showing the reason, cause, and manner of the actors' interaction in the system, use case model can define a system [5, 10].

Use case model revolves around a scenario that defines a process including the system [1]. Usually use case model is in the form of successive events which is valuable for the user. Use case model defines: (a) the actors of the scenario [1], (b) the aims of the actors [10], (c) the related events and the prerequisites of the processes like the start time of the process [10], (d) the main flow of the events and the probable alternatives (the manner of process appearance according to the expression of the actors and system activities [1], and (e) the final manner of the system based on the pre-conditions [10].

### 1.2. Model Structure

Developers utilize use case models primarily to collect performance necessities focusing on the practical conditions and the tasks users intend to do with the information systems [2]. In order to get to know the symbols used in this system, its syntactic structure has been defined in Table 1, according to Unified Modeling Language (UML) version 1.5 [1, 2, 11]. In this concept a system is considered as a close frame of behavior and the user cases are expected to be pattern-oriented and contextual while remaining stable during the lifespan of the information system in the semistructured form.

The elements of this table model the interactions of external users and system and display the behavioral aspects of the system [11]. This is also a starting point for all other diagrams explaining the requirements, architecture, and implementation of the system. Another advantage of the systematic approach based on use case modeling is that the implementation stages can be distinguished from the generation stages [2].

## 2. Methods

The application of use case modeling in analyzing and designing health information systems was examined using electronic Databases (Pubmed, Google scholar) resources, and the characteristics of the modeling system and its effect on the development and design of the health information systems were analyzed.

## 3. Results and Discussion

### 3.1. Use Case Modeling in Health Issues

Despite the high costs of health system, care quality is not satisfactory even in the developed countries [8, 12]. 50% of care specially regarding chronic diseases is offered with beneficial and suitable techniques and unsuitable care accounts for more than 30% [13]. The first problem is that health care organizations usually use care models that show a suitable reaction to acute issues but have been designed for mild chronic diseases. In common care models doctors should focus on the short checkups of acute problems and spend most of their time for chronic patients. Care follow-up is weakly coordinated and communication and information setbacks are common issues. Also, most patients lack the key required skills and information for the self-management of their disease [13, 14].

One of the appropriate methods to improve care is using “collaborative” methods utilizing use case modeling [3]. These care models use team “longitudinal” method and organize care again so that cooperation between an active, aware patient and a ready, equipped care team is improved [10]. In fact use case models combine the patients' information, clinical care management, and remedies based on the evidences to improve the patient's results [7]. Therefore, considering the importance of using use case modeling in analyzing and designing information systems of health section and clarifying the discussion, four executive approaches of this technique in developing and analyzing them follow here.

### 3.2. Use Case Modeling in Imaging Systems

MammoGrid Project is as follows: the goal of the MammoGrid project was to research the possibility of developing a European mammograms information base with access to the software of recent networks in a way that a collection of health care functions can be activated using this base and the networks are equipped in a way that can support the cooperation between health experts all over the European Community. “Use case mode” has been expanded as a shared model to connect the MammoGrid end users, especially doctors, and the development team. This method proves its value in connecting and validating the users' requirements in a repeated, controlled and rising manner [5].

Limitations reported in this project include inference requirements, features and validation, limited time for the experts, the geographical distance between different beneficiaries, and, in fact, the periodic nature of the meetings. During the visits which take several days the experts in this field attend the meetings quite shortly and frequently. Also software engineers in this project paid lesser attention to particular mammography problems and breast cancer screening before. In addition, the requirements team must eliminate the distance between radiologists, network experts and medical image processing experts so that using Computer-Aided Design (CAD) they can collaborate with each other in their own specialized fields. Therefore, the image of this triangular set interaction in developing these use cases and the related evidences is presented. Software experts and Udine radiologists, Torino, Cambridge and medical imaging experts of Mirada and CAD experts of Pisa and Sassari cooperated with each other. The requirements engineering process is used to infer and analyze the needed requirements. The definition and characteristics of the requirements should be developed and validated [6].

Since the main medical participants have quite different goals of their own roles in this project, gradual evolution method allow each of them to absorb the expressed requirements of the other one and expand it in a detailed model in his professional field. As a consequence, events of the subject range and the relations between them appear and evolve leading to natural conceptual object model for the MammoGrid (Table 2) [5, 6].

The principal output of this project is a software platform based on the strengths of the network called MammoGrid Information Infrastructure which integrated several mammography data bases to help doctors develop jointly and reach a joint approach to the analysis of mammography [5].

Teleservices and Remote Medical Care System (TRMCS): TRMCS [15] is one of the other projects to develop health information technology which utilizes use case model in the process of analyzing and designing systems. This study [15] presented a collection of the strengths and weaknesses of UML system as the language of semistructured requirements description, following a realistic approach the software problems, their nature and the way they must be dealt with have been discussed and examined in details. The researcher and his colleagues offer practical actions in another essay so that the critical-qualitative method and the details of different issues are considered in UML shortages especially regarding use case models and system analysis. He calls an objective-based designing method ADOR (Analysis and Design of Requirements Architecture) which includes a hierarchy of abstract objects, each a combination of the user's aspects of structure, implementation capability, behavior, and interaction. In fact, designers utilized their experience of UML in order to eliminate some of the mentioned shortages and developed use case modeling [5, 6, 10]. When the objects are particularly real, it might be supposed that clearly reading UML models is more appropriate than other operational models [5].

### 3.3. The Application of Use Case Modeling in EBR

The role of Evidence Based Medicine (EBM) has increasingly risen in today's health environment. Evidence Based Medicine includes the correct application of external evidences and witnessed information which are combined with clinical experience and specialized priorities of patient so that considering the care of each patient a proper decision is made [10]. The classical definition of Evidence Based Medicine is “a method for health care activities in which the experts are aware of the evidences based on clinical activities and their strengths” [4].

Evidence Based radiology (EBR) is a modern method of radiology based on the medical principals and evidences which is used particularly in the fields of diagnostic imaging and interference radiology [3, 5]. In addition, imaging is an effective method for screening, documentation of the existence or lack of a disease or special conditions, and managing many medical conditions. Also imaging studies and EBR can form an important framework for the medical activities based on evidences [10].

Use case modeling is used in different environments to evaluate data access conditions, improve radiological advice, and support EBR. In a study carried out by one of the researchers, 5 clinical test sites were examined to consider the needs of surgery, clinic, and radiology experts. These sites included 3 fields of radiology (chest radiology, neuroradiology, and pediatric radiology) and 2 outpatients' clinic (pediatric urology and adults' lung/medical center) [10]. Staff and graduated students in each field helped doctors and recorded key components of daily activities. Six members of chest Radiology College examined the interpretations, 4 of them worked in pediatric radiology ward and 1 was in neuroradiology ward. The manager of each clinic supported the project in pediatric urology and lung/medical wards, the manager also interacted with nurses and other staff [4]. The study at hand shows that the system should first supply access to several clinical data resources like Radiology Information System (RIS), HIS and PACS so that the patient's data can be accessed and utilized easily and contribute to the comprehensive radiologic advices [2]. Secondly, quick access to the external evidences regarding the patients with similar conditions and also medical resources is ensured and EBR is supported. With the help of other methods these requirements can be extended to other organizations and take into account the special conditions required for the data base [2, 4].

The present study which utilizes use case modeling to determine EBR requirements in the field of radiology points to the limitations as well. In order to fully process the model in clinical and information environments of the radiologists more activities are required so that the requirements of these users are determined. In addition, an official evaluation of the results must be administered so that the manner of EBR effect supporting the electronic infrastructures on the care process of the patient is determined [10].

### 3.4. Analysis of the Model's Characteristics and Operational Functions

Use case model can be utilized in all methodologies with an objective-oriented approach specially phase development [1]. Use cases have been used in the studies as the most important system behavior definition modeling tool and in order to define the manner of user's interaction with the system to perform health information systems activities [5, 10, 16] and the notable point in this model is its accordance with health care services characteristics from the viewpoint of focusing on an activity in a given time [17], Table 3 clearly deals with the characteristics and effects of it [1, 4, 10, 11, 16, 17].

One aspect of use case traditionally ignored but beneficial dealing with the sensitive data (like patients information) is security concerns which emphasize the infrastructure of the system. next point to consider is integrity and unity of the information. In this case the patient/user will be the actor of the use case because he is innately interested in controlling his own data [14].

## 4. Conclusion

For the medical imaging information systems in which staff select and add the information to the files, clinical data direction must be modeled in a particular case to filter and present suitable information to the medical imaging expert. Utilizing use case technique and intelligent classification and combining and summarizing the patients' data, temporary and inducing relations between important medical events can be established and the doctor's task in using medical information will be easier [10, 18, 19].

Discussions related to use cases are the related scenarios, data requirements and communications lead to a proper definition about final models which stabilize clinical activities in an effective way [13]. In the studies carried out, since the participants in the design and analysis of the models had quite different goals in their own projects, the gradual evolution and interactive method modeling approach allow each of them to absorb the requirements expressed by others and this can lead to the professional development of each of the participants and the objects in the range of the issue being studied and the relation between them will become more clear.

These experiences show that the progress in the development stages is strengthened through gradual evolution and repeated user requirements. This can reduce the requirements engineering process cycle in large projects [20] and this is assigned to use case model approach in directing the problem towards understanding, focusing on the start and analysis, better planning, control, and repetition [10, 13].

The point more apparent in MammoGrid study is using rational architecture model in describing its requirements, recognizing the systems and main components and determining the relation between them and the characteristics of the intermediating agents [5, 6]. This determines the provident detailed system design and provides the regular analysis of use case scenarios, data flows, and architectural activities between the systems.

In contrast, the challenge faced in utilizing modeling technique of care processes studied is the lack of an exact plan of system integrity ensuring processes so it will confuse model designers and will have problems in recognizing start point, manner of starting the process and consequently the quality of preliminary design, health care services or information systems and also the security of the data and systems will not be satisfactory. Therefore, using proper suitable analysis models of quality assurance will prevent deviations in the architecture of the systems in the medical imaging information systems designing and in a way will make the tracing of issues in the process of designing possible.

Next, deficiency is the pure evaluative methods of analytical models which do not seem to be considered as an independent subject and is presently one of the serious problems in health section software development [21, 22]. In case enough attention is paid to this issue in the models architecture planning, the results will be valuable for improving information systems modeling process.

On the whole, efforts have been made to deal with a set of innovations, design requirements engineering challenges and creation of analysis models and conceptual design of information and communication structures of medical imaging systems. Generating more extensive and ordered models will be possible by following these and performing a deeper analysis of modeling relations. Use case approach needs deeper and more serious studies for modeling health care information processes and developing systems providently. If more attention is paid to deeper analysis of modeling relations, it can surely be an effective, proper tool to display health information system range and enhance the relations among system analyzers, developers, and users.

## Figures and Tables

**Table 1:** The syntactic structure of use case model.

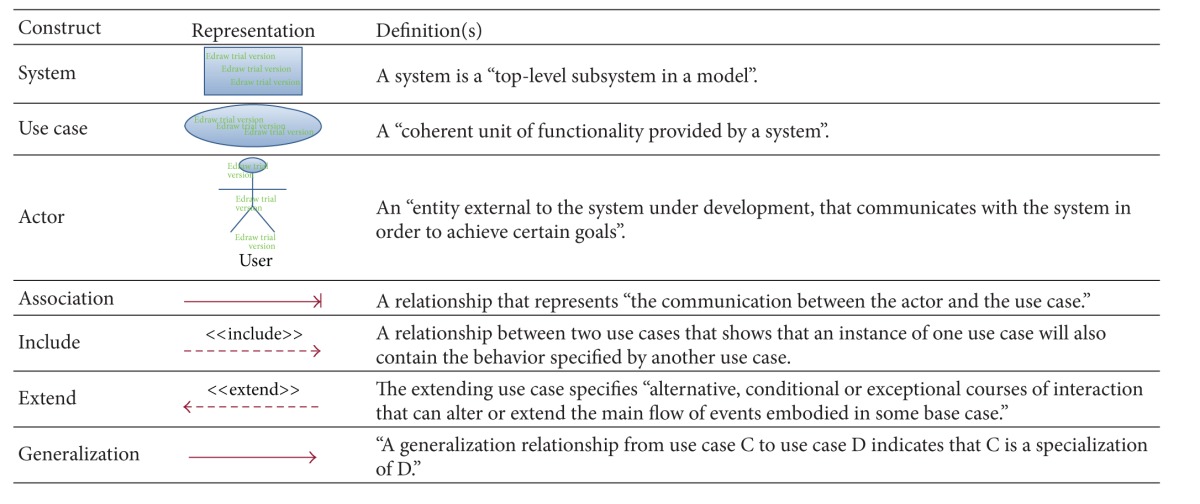

**Table 2:** Dimensions of the model with the use case function of radiologist analysis.

Key dimensions of use case modeling	Use case function of radiologist analysis
Actor	Mammogram analyst

Preconditions	User-authentication

Nonfunctional requirements	CAD software interface requirements

Flow of events	(1) as per selection of the mammogram analyst to (2) link to the appropriate extension point below

Extension points	(1) View mammogram and patient details (2) Annotate mammograms and patient details (2.1) Diagnose study > (2.2) Diagnose series > (2.3) Annotate image > (2.4) Request CAD > (2.5) Link annotations > (2.6) Request CAD in mammogram region (3) Execute radiological queries > (3.1) Formulate radiological query > (3.2) Refine radiological query

Alternative flows	(1) Unsuccessful User authentication (2) CAD interface error (3) Invalid query selections

Postconditions	(1) Mammogram image annotated (2) Patient details changed (3) Results of query execution (grid)

**Table 3:** Use case modeling characteristics and operational functions in medical imaging information systems.

*N*.	“Use case modeling” characteristics	Operational functions
1	A very realistic method for the patients, users and analyzers to have an accurate viewpoint about their own system	(i) Establishing a better correlation between medical imaging information system users, analyzers and developers. (ii) Studying the weaknesses and strengths of the system.

2	Determining the operation framework and protocols	(i) Determining operation language specified conditions (in codes). (ii) Defining operation exceptions. (iii) Defining operation scale and time. (iv) Defining concurrency several control processing. (v) Defining conditions before and after the operation

3	Frequent relations between the units during data flow and health system processes	(i) Objects can be used again in designing medical imaging information systems. (ii) The advantages of having an integrated system.

4	Classes can be used as standard patterns	(i) Redundancy reduction. (ii) Setting new classes quickly. (iii) Developing standards and their compatibility. (iv) Easy support of the exceptions operation may face.

5	Designing the communication and information diagram of the system and parameters	(i) Exchange of information between patients and service providers. (ii) Presenting a clear image of communication and information processes. (iii) Determining performed operations in each time stage. (iv) Determining the factor acting on the system. (v) Determining the range of the project being implemented. (vi) Having a basic mentality of the forgotten activities

6	Possibility of the information system users and analyzers concentration on the manner of the users interaction for performing an individual activity with the system	(i) Better understanding and collecting the requirements of system user. (ii) Better relation between system user and analyzer. (iii) Preparing the ground for better exchange of information between user and system analyzer.

7	Examining the involved information systems from different views	(i) Better understanding and modeling of the users requirements. (ii) More complete recording of medical imaging information system.

8	Constant evaluation, control and filtering of the related information system	(i) Adopting the real requirements the users. (ii) Medical imaging information system with high-quality.
